# Improvement of Terahertz Photoconductive Antenna using Optical Antenna Array of ZnO Nanorods

**DOI:** 10.1038/s41598-019-38820-3

**Published:** 2019-02-05

**Authors:** Mohammad Bashirpour, Matin Forouzmehr, Seyed Ehsan Hosseininejad, Mohammadreza Kolahdouz, Mohammad Neshat

**Affiliations:** 1 School of Electrical and Computer Engineering, College of Engineering, University of Tehran, Tehran, Iran; 2 Department of Electrical Engineering, Yazd University, Yazd, Iran

## Abstract

An efficient terahertz (THz) photoconductive antenna (PCA), as a major constituent for the generation or detection of THz waves, plays an essential role in bridging microwave-to-photonic gaps. Here, we propose an impressive approach comprising the use of arrayed zinc oxide nanorods (ZnO NRs) as an optical nanoantenna over an anti-reflective layer (silicon nitride) in the antenna gap to boost the photocurrent and consequently the THz signal. The numerical approach applied in investigating the optical behavior of the structure, demonstrates a significant field enhancement within the LT-GaAs layer due to the optical antenna performing simultaneously as a concentrator and an antireflector which behaves as a graded-refractive index layer. ZnO NRs have been fabricated on the PCA gap using the hydrothermal method as a simple, low cost and production compatible fabrication method compared to other complex methods used for the optical nanoantennas. Compared to the conventional PCA with a traditional antireflection coating, the measured THz power by time domain spectroscopy (TDS) is increased more than 4 times on average over the 0.1–1.2 THz range.

## Introduction

In the past decade, THz technology and its applications have attracted lots of attention due to the unique properties of THz radiation such as its non-ionizing nature, minimal effect on human body, sensing and spectroscopy of large molecules, and penetration through wide variety of materials (paper, wood, plastic, and fabric)^1^. These important features have made THz technology a great candidate in security-related, imaging, biochemical spectroscopy applications and nondestructive tests, to name a few^2–4^. One of the most common approaches in generating THz waves is utilizing photoconductive antennas (PCAs). In this method, an ultrafast femtosecond laser interacts with a biased photoconductive semiconductor with a sub-picosecond carrier lifetime (low-temperature-grown GaAs (LT-GaAs)) that results in a transient photocurrent. According to the radiation theory, transient photocurrent with a sub-picosecond (ps) pulse width, eventuates to a radiation of electromagnetic waves in the THz spectrum^5^. However, despite PCA advantages such as room temperature operation, compact design, and broadband radiation, it also provides low optical-to-THz conversion efficiency that limits its applications^6,7^.

Many efforts have been devoted to increase the laser pulse coupling into LT-GaAs substrates, and to improve the performance of the PCA; such as using antireflection coating on LT-GaAs^8^, distributed Bragg reflector made of AlAs:AlGaAs stacks located under the LT-GaAs layer^9^, three dimensional nanoplasmonic structure^10^, double layer nanoplasmonic structure^11,12^, recessed electrode and recessed nanoplasmonic array, nano-spaced electrodes^13^, optical plasmonic nanoantenna^14,15^, plasmonic nanostructure^16–20^ and else^21–23^. Most of these methods require electron beam lithography which raises the cost and time of fabrication significantly. Thus, proposing a structure exhibiting superior optical absorption in LT-GaAs without requiring a complex fabrication method is highly desirable. Moreover, despite the advantages of plasmonic nanoantennas like the strong field localization, they also present large dissipative losses. This limitation can be overcome by the use of a non-metallic optical nanoantenna that has much higher damage threshold due to its low dissipative loss^24^. Furthermore, in order to reduce the Fresnel reflection of a femtosecond laser from the surface of LT-GaAs, as in other optoelectronic devices e.g. solar cells^25^, photodetectors^26^, or light emitting diodes^27^, the antireflection coating (ARC) plays a critical role. The main limitation of a single ARC layer is that the reflected light does not get a second chance of incidence, therefore using a nanostructured layer as a graded-refractive index layer is much appreciated^17,28^. Recently, Gric *et al*. have simulated numerically different transparent-conducting oxides (TCOs) nanocylinders with different diameters to enhance the absorption in the PCA photoconductive layer and improve its performance. They have introduced and optimized the geometrical parameters of AZO, ITO, TiN and ZrN nanocylinders as an optical nano antenna to enhance the terahertz radiation of PCA as a plasmonic nanostructure^29^. According to their results, TCOs show better performance in comparison with metallic nanostructures.

In this work, an array of ZnO nanorods (NRs) has been employed on the PCA gap as an optical concentrator and an antireflective coating at the same time which has never been done before. On the one hand, the ZnO NRs act as an optical nanoantenna which boost the local field and optical absorption of femtosecond laser pulse in the LT-GaAs layer. On the other hand, ZnO NRs act as a virtual layer with spatially varying refractive index between air, silicon nitride and LT-GaAs to minimize the reflection from the surface. Consequently, the optical power enhancement results in more photocarrier generation, and so higher power for terahertz emission.

## Device Structure and Design

**Figure 1:**
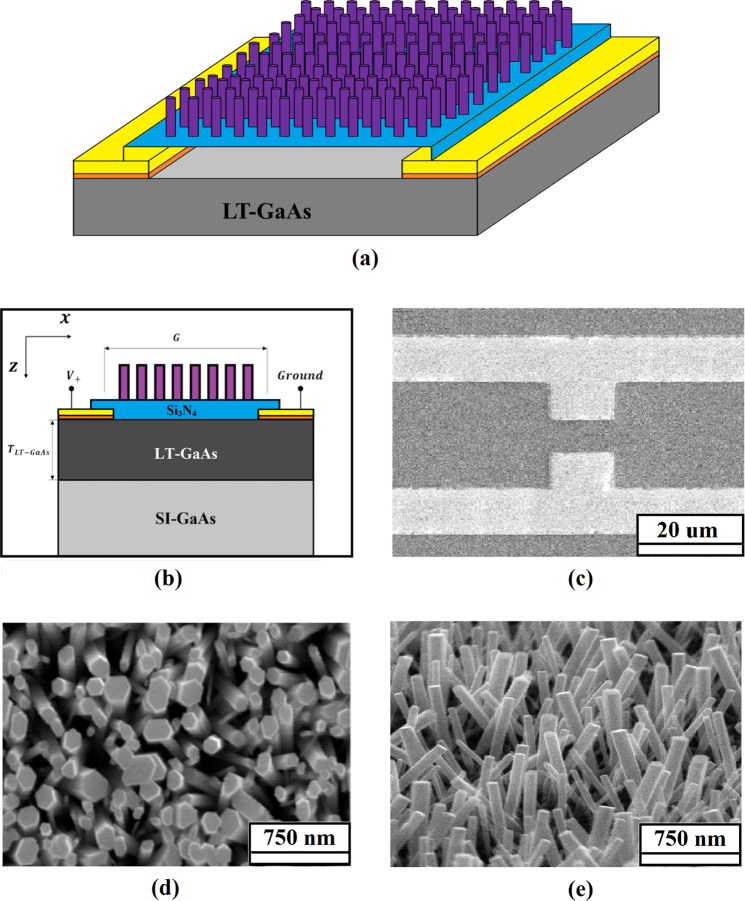
(**a**,**b**) Schematic diagram of the proposed PCA (three dimentional and front views), FESEM image of (**c**) PCA, (**d**) ZnO NRs (top view) and (**e**) ZnO NRs (cross-sectional view).

Schematic views of the proposed PCA are shown in Fig. 1a,b. An LT-GaAs layer with 2 µm thickness on the semi-insulating GaAs (SI-GaAs) was used as the substrate. The Dipole antenna arm (/electrodes) were 10/150 nm thick Ti/Au layer with a 5 µm gap. The Titanium layer was used as the gold adhesion promoter in this design. The width and length of the dipole gap and the total length of the antenna are 5 µm, 10 µm, and 40 µm, respectively. A silicon nitride layer with a thickness of 100 nm was used as an ARC to minimize the reflection from the surface. Moreover, Si_3_N_4_ isolated the gap of the antenna from the ZnO NRs. Finally, ZnO nanorods with an average diameter of 120 nm and height of 700 nm were grown on the Si_3_N_4_ layer.

Field emission scanning electron microscopy (FESEM, HITACHI S-4160) images of fabricated PCA and ZnO NRs are illustrated in Fig. 1c–e. X-ray diffraction (XRD) by Philips X’pert, with a Cu-Ka radiation (λ = 0.15418 nm) was used to explore the crystallinity of the ZnO NRs. The as-synthesized products were also characterized by transmission electron microscopy (TEM, JEM-2010-JEOL, Japan, 400 kV). The field emission studies were carried out in a chamber with a vacuum of ~1.33 Pa at room temperature.

**Figure 2:**
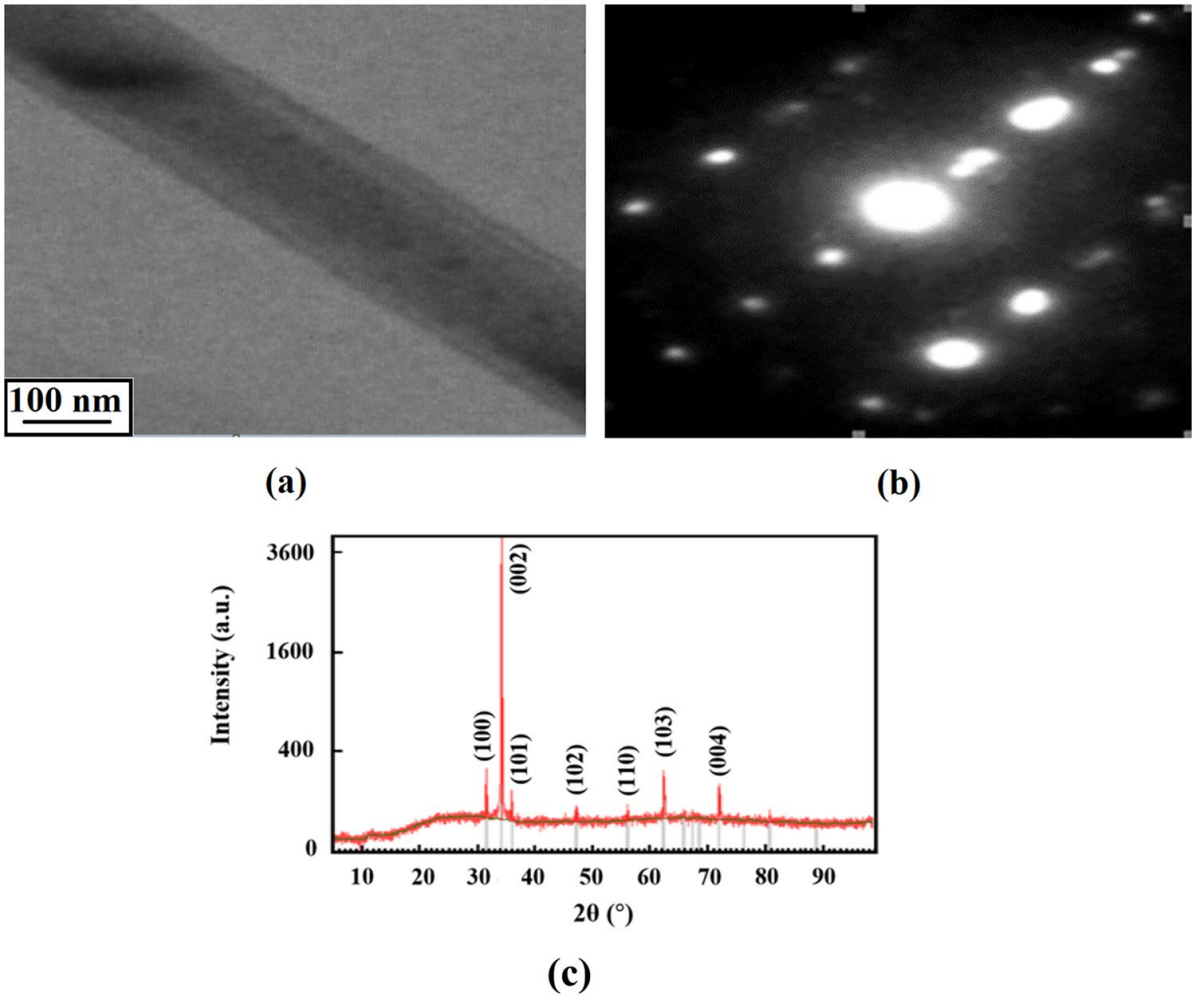
(**a**) TEM image of ZnO NRs, (**b**) the selected-area electronic diffraction pattern (SAED) of a typical ZnO NR and (**c**) X-ray diffraction (XRD) of ZnO NRs.

The TEM image of the grown ZnO NR indicates that high quality crystalline NRs with a hexagonal shape and an average diameter of 120 nm have been successfully grown (see Fig. 2a). Figure 2b also illustrates a typical NR’s selected-area electron diffraction pattern. This spot-like pattern is sufficient evidence that the ZnO NRs were grown along the c-axis orientation at low temperature (90 °C). Figure 2c shows the XRD pattern of ZnO NRs in which the positions of all peaks are in accordance with JCPDS Card No. 36–1451, indicating that all NRs have wurtzite crystal structures. The high relative intensity of (002) peak denotes the preferential growth in the basal direction^30^.

## Results and Discussion

A solver based on the Finite element method (FEM) was applied to investigate the electrical and optical behavior of the proposed structure through combining Maxwell’s equations with the drift-diffusion/Poisson equations^31^.The numerical results show that the calculated photocurrent of the proposed structure experiences a 2.5-fold and 6-fold increase compared to the conventional PCA with and without ARC, respectively (Fig. 4a). To understand the optical advantage of NRs over the anti-reflector layer for a THz PCA, let us study the backward reflection and the local field enhancement of the optical pump beam.

The reflectance (R) of an incident light from the interface of any two media with the refractive index of *n*_1_ and *n*_2_ for a normal incident beam can be calculated by Fresnel equation:

1 $$R={|\frac{{n}_{1}-{n}_{2}}{{n}_{1}+{n}_{2}}|}^{2}$$

**Figure 3:**
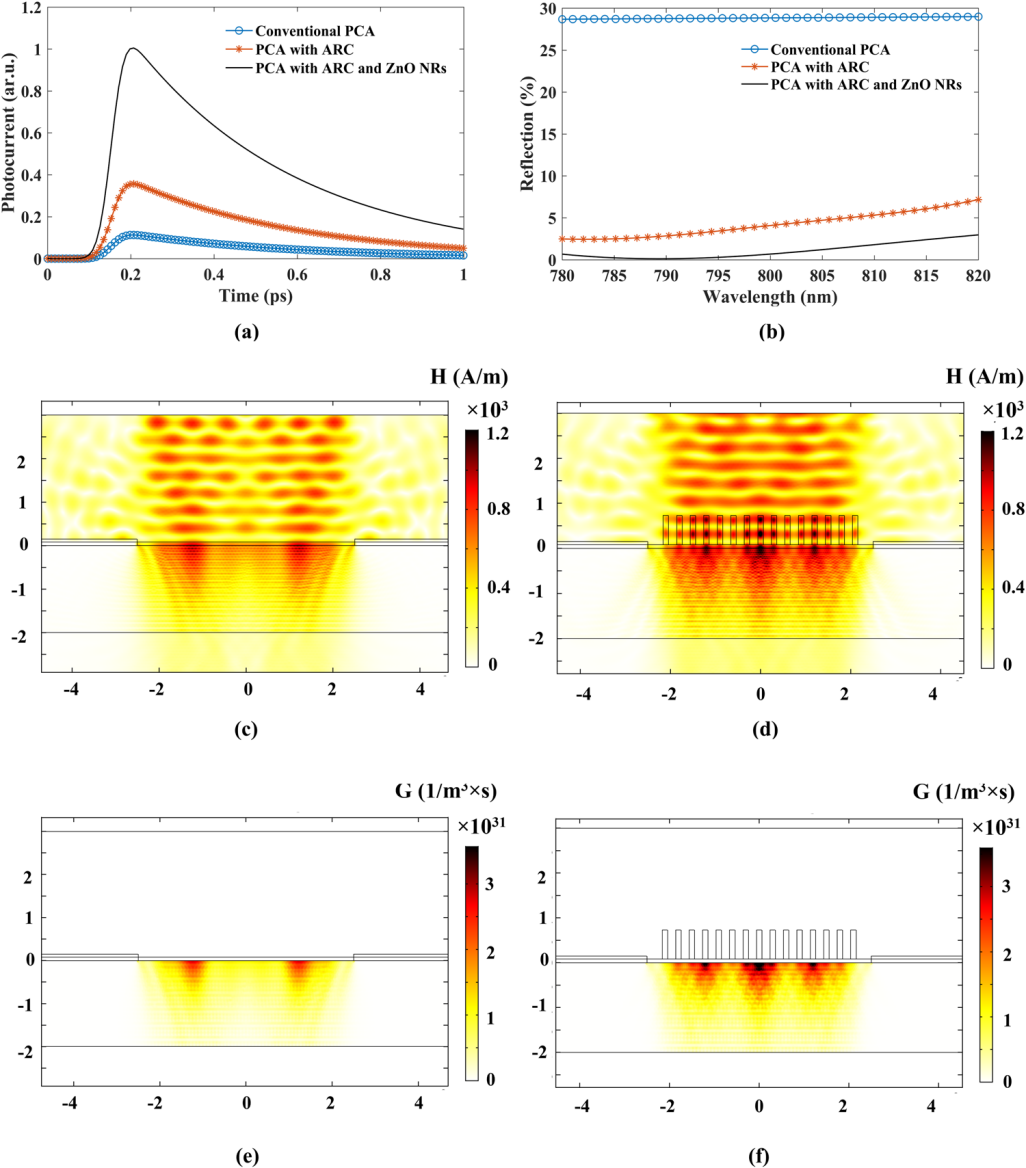
Comparison of the simulated (**a**) photocurrents as a function of time and (**b**) reflections as a function of optical wavelength of conventional PCAs without and with ARC and the proposed PCA, The Simulated magnetic field distribution and generation rate of (**c**,**e**) conventional PCA with ARC and (**d**,**f**) the proposed PCA, respectively.

In order to minimize the reflection of the desired wavelength from the interface of air and substrate, a non-absorbing layer with a thickness of ¼ wavelength (d_ARC_) of the incident light and a refractive index (n_ARC_) equal to the square root of the substrate’s refractive index, is required^32^. The major problem of a single-layer quarter-wavelength ARCs in PCAs is finding a non-absorbing material with the desired refractive index (*n* = 1.9) between air (*n* = 1) and LT-GaAs (*n* = 3.67). One promising solution to this limitation is surface texturing with a cross-sectional dimension less than the incoming light wavelength. In this case, the structured surface acts as a medium with spatially varying refractive index. Consequently ZnO NRs on the Si_3_N_4_ layer can act as an effective ARC on LT-GaAs to remarkably improve the PCA’s performance. The simulation result of the power reflection coefficient is shown in Fig. 3b. Considering 800 nm as the fs laser wavelength, using ZnO NRs reduces the reflection to less than 0.8% compared to the 4% of the PCA with Si_3_N_4_ ARC^8^ and the 29% of the conventional PCA without ARC^31^. According to the shape and length variation of ZnO NRs, they can be considered as a graded refractive index layer.

**Figure 4:**
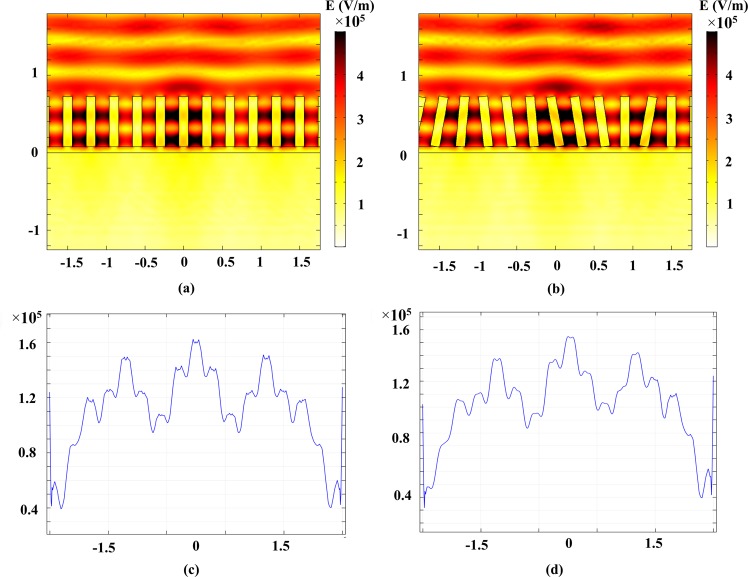
The electric field distribution of (**a**) aligned ZnO nanorode (**b**) random orientation ZnO nanorode, Electric field cutline at 30 nm under the surface of LT-GaAs layer for (**c**) aligned nanorode (**b**) random orientation nanorode.

The simulated magnetic field distributions of the PCA without and with ZnO NRs are depicted in Fig. 3c,d, respectively. It can be found that the array of NRs acts as a dielectric nanoantenna with the ability to create a strong local field enhancement by concentrating the laser pulse. Consequently, the optical power absorbed by the semiconductor along with the generation rate, increase. According to Mangalgiri *et al*.^33^ theoretical study, low dielectric NRs like ZnO have high scattering ability and show excellent antenna like resonances when nanostructured. This property can be used to couple the 800 nm laser pulse to LT-GaAs layer. We have assumed that each photon that absorbed by the photoconductive layer generates an electron-hole pair. So, the time dependent carrier generation rate (G) has been calculated as:

2 $$G(x,y,z,t)=(4\pi {k}_{PC}/hc){P}_{s}(x,y,z)\exp (4\,\mathrm{ln}(0.5)\frac{{(t-{t}_{o})}^{2}}{{D}_{t}^{2}})$$

Where, *k*_*PC*_ is the imaginary part of refractive index, c is speed of light, *D*_*t*_ is laser pulse duration, h is Planck’s constant and *P*_*s*_(*x*, *y*, *z*) is total power flux density that can be obtained by Maxwell’s equation solving. Figure 3e,f show the distributions of generation rate (G) in the substrate with and without NRs. A quick exploration reveals that the generation rate inside the GaAs in the presence of an optical antenna is significantly larger than without NRs. Using ZnO NRs reduces the reflection of 800 nm laser beam to less than 0.8% from the LT-GaAs surface and enhances the local field near the surface of LT-GaAs layer by concentrating the optical beam. Higher field results in higher magnetic field inside the LT-GaAs and so more power flux density and according to Eq. 1, more generation rate. The generation rate enhancement results in transient photocurrent increase (Fig. 3a) and so terahertz radiation.

One major challenge of ZnO NRs growth is random orientation of each nanorode that can degrade the performance of PCA. To investigate the effect of random orientation of ZnO NRs on PCA performance, we have applied random tilt of 1–20% from the aligned NRs and have compared the electric field distribution of aligned ZnO NRs and ransom orientation ones. In Fig. 1a,b, the electric field distribution in the LT-GaAs layer is shown. As can be seen, ZnO NRs random orientation does not have so much effect on electric field distribution. According to Fig. 1c,d, the peak of electric field at 30 nm from the surface of LT-GaAs for aligned ZnO NRs is only 6% higher than random orientation NRs. Random orientation may increase the 800 nm laser pulse reflectance from 0.8% to more than 2% but It does not decrease the photocurrent in considerable value and so the terahertz radiation. Because, the dominant reason for photocurrent enhancement is local field enhancement in the LT-GaAs because of ZnO nanorode. They act as an optical nano-antenna and concentrate the laser pulse at LT-GaAs layer surface and so the generation rate near the surface increases and results in higher photocurrent and so higher terahertz radiation.

**Figure 5:**
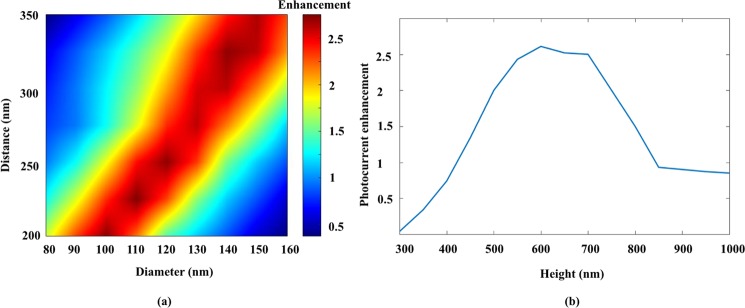
PCA transient photocurrent enhancement as a function of (**a**) ZnO NRs diamter and center to center distance and (**b**) ZnO NRs height.

In order to obtain the best performance of PCA, the ZnO NRs diameter, center to center distance and height effect on PCA performance was investigated numerically. First, the height of nanorodes set to 650 nm and the diameter (D) swept from 80 nm to 160 nm for the center to center distance (P) varying from 200 nm to 350 nm. The enhancement of photocurrent as a function of NRs diameter and distance respect to conventional PCA with Si_3_N_4_ antireflection coating is shown in Fig. 5a. As can be seen, there is 2.5-fold photocurrent enhancement in some area. In the next step, the NRs diameter set to 120 nm and distance to 250 nm (according to our fabrication ability) and the simulation were done for different heights. The highest enhancement obtained at H = 600 nm. As can be seen in Fig. 5b, the height of 550 to 700 nm is a good choice for ZnO NRs height for optimum performance.

**Figure 6:**
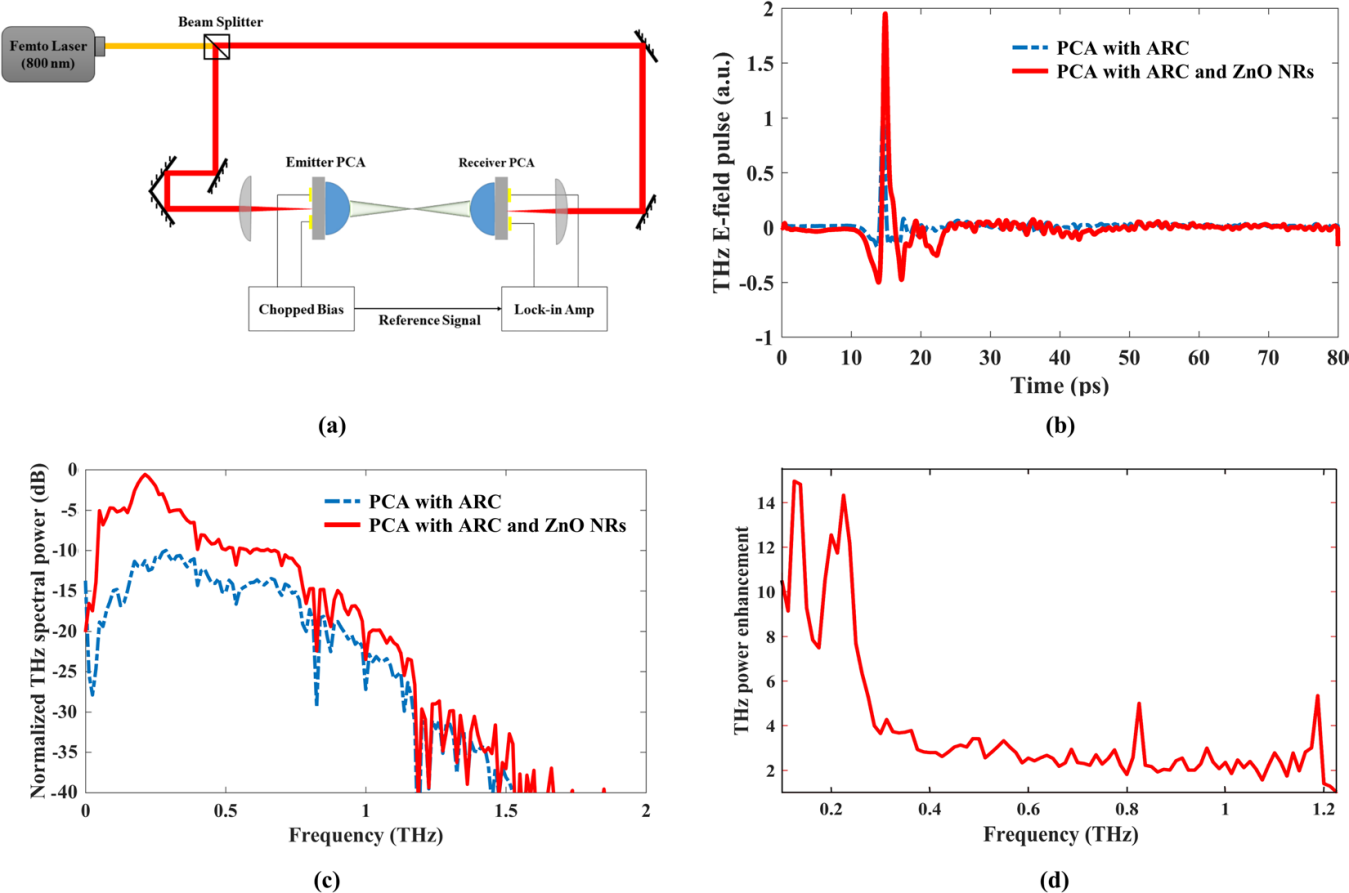
(**a**) THz a) E-Field as a function of time and (**b**) THz power spectrum comparison of PCA with ARC and the proposed PCA, and (**c**) The experimental setup for measuring THz generation using an 800 nm femtosecond laser (**d**) THz power enhancement of the proposed PCA compared to the conventional PCA with ARC at different frequencies.

A pump-probe THz measurement kit from TeTechS Inc. was used to characterize the radiated THz signal of the fabricated PCAs. For this purpose, an 800 nm fiber laser with the width of 100 fs pulse, repetition rate of 80 MHz an average power of 14 mW on the emitter side and 10 mW on the detector side was used as an optical excitation source (Fig. 6a). The applied bias voltage on transmitter was ±10 *V* square wave. In order to obtain the highest THz signal from the PCA, the optical pump was tightly focused upon each device. Designed and fabricated PCAs were mounted on the hyper-hemispherical silicon lens to collimate and guide the generated THz signal out of LT-GaAs. The fabricated PCAs were used as an emitter while the detector was a commercial dipole PCA with the gap length of 5 µm and the total length of 20 µm from TeTechS Inc.

Zinc oxide has higher conductivity in comparison with LT-GaAs. So, to avoid current path from ZnO insteat of LT-GaAs, the Si_3_N_4_ layer was used to isolate the LT-GaAs layer and antenna electrodes from ZnO NRs. Silicon nitride layer prevents electrical contact between ZnO NRs and PCA electrodes, so the dark resistance will not change comparing with conventional PCA. The dark resistance of fabricated PCAs (conventional PCA and optimized PCA with ZnO NRs) were 20 ± 2 MΩ. The measured time domain waveform for the reference PCA with a Si3N4 ARC layer and the proposed PCA with ZnO NRs are presented and compared in Fig. 6b. As can be seen, THz electric field peak shows a 2-fold enhancement compared to the reference PCA. THz power spectrums of both antennas are illustrated in Fig. 6c. THz waves with the spectral ranging between 0.1–2 THz and a dynamic range of 65 dB were obtained using an 800 nm femtosecond laser. The measured THz power by time-domain spectroscopy (TDS) is increased more than 4 times in average over the 0.1–1.2 THz range compared to the reference one. Figure 6d illustrates the THz power enhancement of the proposed PCA with ZnO NRs compared to the conventional PCA with ARC at different frequencies. The uneven power enhancement could be associated with the variation of the gap impedance due to the loaded NR array^15^. In a similar research, metallic nano-island as an optical nanoantenna have been proposed by Park *et al*. that results in 2 times THz power emission compared with conventional PCA. They have used thermal dewetting of silver thin film to create plasmonic nano antenna. Silver nano-islands in PCA gap excite the surface plasmon and enhance the terahertz radiation^34^. In this work, we have used ZnO NRs (non-metallic nanostructure) as an anti-reflector and optical nanoantenna to concentrate the laser beam near the surface of LT-GaAs. This will lead to local high field near the surface of LT-GaAs layer and so more photocarrier will generate. Higher photocarrier results in higher photocurrent and so higher terahertz file radiation. Higher generation rate causes more transient photocurrent and so more terahertz radiation that results in two times higher THz electric field pulse emission compared with conventional PCA.

In summary, a novel idea of integrating optical nanoantennas based on dielectric NRs to improve the performance of THz PCA has been presented in this study. The effect of ZnO NRs on the THz PCA performance was investigated by a numerical approach and verified by the experiments. According to the simulation results, the photocurrent has been remarkably enhanced in the proposed structure due to the decrease of backward reflection of the optical pump and the increase of local fields. The measurement of the fabricated PCA in THz time-domain spectroscopy set up showed a THz power enhancement of more than 4 times on average over the 0.1–1.2 THz range. Therefore, ZnO NRs can be a great breakthrough for fabricating low-cost high-performance THz photoconductive sources.

## Method

### ZnO decorated photoconductive antenna fabrication Process

In order To fabricate this device, LT-GaAs wafers, from BATOP Company, were primarily cleaned using a standard process. Thereafter, the fabrication process began with patterning a dipole antenna using photolithography, followed by Ti/Au (10/150 nm) sputtering. Next, the dipole structure was fabricated by lift-off process in acetone. Then, a 100 nm Si_3_N_4_ as an ARC, passivation and isolation layer was deposited by plasma enhanced chemical vapor deposition. ZnO nanorods were grown with pre-deposition of a 20 nm aluminum-doped ZnO (AZO) seed layer. The NR growth was performed hydrothermally in a chemical bath solution containing 25 mM zinc nitrate hexahydrate (Zn(NO_3_)_2_.6H_2_O, Merck) and 25 mM hexamethylenetetramine ((CH_2_)_6_N_4_, Merck). The growth process occurred at 90 °C in 2 hours which resulted in NRs with a length of about 700 nm and a diameter around 120 nm. As the last step, the ZnO NRs and the Si_3_N_4_ layer were etched on the PCA’s bias pads in order to access the electrode pads. In order to open electrodes widow, first, thick photoresist was spin coated on ZnO NRs to cover all the NRs. After that, electrodes window opening was performed by patterning the device by standard photolithography followed by immersing in to the diluted HCL for ZnO NRs etching. In the next step, BHF was used to etch the Si_3_N_4_ layer. Finally, the photoresist is removed by sonication of PCA sample in the acetone solution.
